# Toward a Deeper Understanding of the Genetics of Bipolar Disorder

**DOI:** 10.3389/fpsyt.2015.00105

**Published:** 2015-08-03

**Authors:** Berit Kerner

**Affiliations:** ^1^Semel Institute for Neuroscience and Human Behavior, University of California, Los Angeles, Los Angeles, CA, USA

**Keywords:** bipolar disorder, deep sequencing, genetic models of transmission, rare variants, common genomic polymorphisms

## Abstract

Bipolar disorder is a common, complex psychiatric disorder characterized by mania and depression. The disease aggregates in families, but despite much effort, it has been difficult to delineate the basic genetic model or identify specific genetic risk factors. Not only single gene Mendelian transmission and common variant hypotheses but also multivariate threshold models and oligogenic quasi-Mendelian modes of inheritance have dominated the discussion at times. Almost complete sequence information of the human genome and falling sequencing costs now offer the opportunity to test these models in families in which the disorder is transmitted over several generations. Exome-wide sequencing studies have revealed an astonishing number of rare and potentially damaging mutations in brain-expressed genes that could have contributed to the disease manifestation. However, the statistical analysis of these data has been challenging, because genetic risk factors displayed a high degree of dissimilarity across families. This scenario is not unique to bipolar disorder, but similar results have also been found in schizophrenia, a potentially related psychiatric disorder. Recently, our group has published data which supported an oligogenic genetic model of transmission in a family with bipolar disorder. In this family, three affected siblings shared rare, damaging mutations in multiple genes, which were linked to stress response pathways. These pathways are also the target for drugs frequently used to treat bipolar disorder. This article discusses these findings in the context of previously proclaimed disease models and suggests future research directions, including biological confirmation and phenotype stratification as an approach to disease heterogeneity.

## Introduction

“Manic-depressive illness magnifies common human experiences to larger-than-life proportions” (1). This opening sentence to Goodwin and Jamison’s acclaimed and comprehensive book on bipolar disorder places the often extreme and strange-appearing symptoms of mania and depression in a more comprehensible framework of shared human experiences. Bipolar disorder is a severe, complex psychiatric disorder, but still, it is so common that most people likely know a friend, a neighbor, or even a family member affected with this disease. After all, with an estimated prevalence rate of 2.4% (2) and a world population of 7 billion people, it is expected that several million patients might suffer from bipolar disorder worldwide. The core symptoms of bipolar disorder are episodes of abnormally elevated, expansive, and irritable mood accompanied by inflated self-esteem and grandiosity. Decreased need for sleep, increased talkativeness, and flight of ideas, could also be present, in addition to excessive goal-directed activity and extreme involvement in pleasurable activities, which frequently are associated with a high potential for painful consequences (3). To meet diagnostic criteria, the symptoms must have caused marked impairment in social and occupational functioning or required hospitalization to prevent harm to oneself or others. Symptoms of depression can precede or follow manic episodes, and sometimes even accompany manic episodes, although they are not required for making the diagnosis. Psychotic symptoms, such as hallucinations and/or delusions, occur in about 50% of bipolar disorder patients, suggesting some symptomatic and even pathophysiological overlap with schizophrenia (4). For many patients, the mood symptoms can be so agonizing that suicide seems to be the only escape (2). Bipolar disorder appears to have strong genetic risk factors. Twin studies have suggested a monozygotic concordance rate of 0.43, and population-based family risk studies have estimated a heritability rate of about 58% (5, 6). Environmental risk factors, such as trauma (7, 8), infection, and inflammation (9), have been found to contribute to a lesser degree. Despite the widespread occurrence, the cause of the disease remains elusive.

KEY CONCEPT 1. Single gene Mendelian transmission Many rare, Mendelian disorders, such as cystic fibrosis, are caused by mutations in one or a few genes. The disease manifests if one allele (dominant inheritance) or both alleles (recessive inheritance) of a gene carry the mutation and environmental influences are of minor importance.

KEY CONCEPT 2. Multivariate threshold model This genetic model is concerned with quantitative traits that are conceptualized as being normally distributed in the population. A change in the phenotype occurs if a threshold of genetic or environmental influences has been reached. The relationship between traits and disease is often unclear.

KEY CONCEPT 3. Oligogenic quasi-Mendelian mode of inheritance This genetic model conceptualizes disorders as being influenced by a small number of genetic mutations in genes that are potentially related to a specific biological function. A disorder occurs if the interacting mutations are present and environmental factors are considered of minor importance. The inheritance pattern of the disease follows Mendelian rules if all mutations are inherited jointly.

KEY CONCEPT 4. Genetic risk factors The genetic code contains information about the structure, function, and timely expression of proteins, which are the basic building blocks of the cell machinery. Changes in the base-pair sequence of this code (mutations) could lead to disease-causing changes in the structure and/or function of the encoded proteins. The protein expression level or timing might also be altered.

## Early Models of Disease Transmission and Heritability in Bipolar Disorder

In some families, bipolar disorder has been transmitted over several generations, closely resembling a Mendelian disorder (10, 11). This observation had originally inspired researchers to study rare, large multi-generational pedigrees under the assumption of a single gene with large effect size and autosomal dominant, recessive, or X-linked inheritance (12, 13). After initial enthusiasm supported by strong genetic linkage signals, it was quickly discovered that these results could not be replicated (14). Incomplete penetrance, etiological heterogeneity, and recombination events might have contributed to the replication failure. However, it was also likely that the underlying disease model was not supported by the data. After all, not all segregation studies had supported a disease model built on a single major disease locus (15–18). The high frequency in the population of bipolar disorder also clearly distinguished the disease from rare Mendelian disorders. As a consequence, the idea of a single major risk locus was quickly rejected (19, 20).

## Why is Bipolar Disorder so Common in the Population?

**Figure 1:**
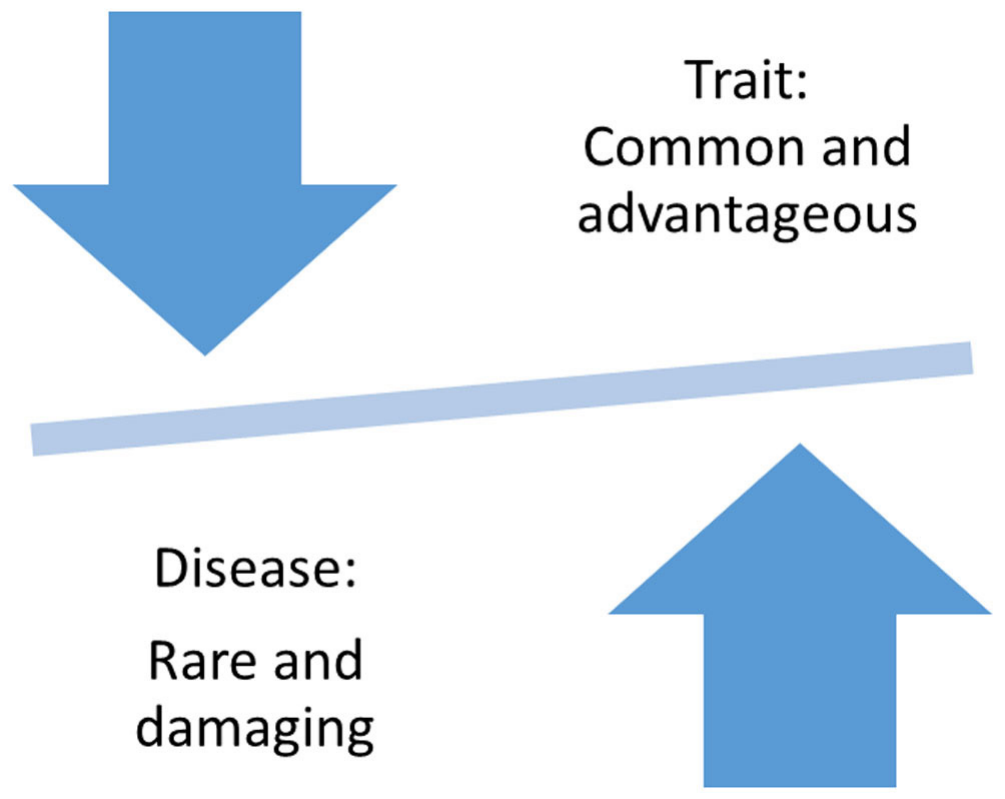
**Evolution-based hypothesis about traits and diseases**.

The question of why and how severe and debilitating disorders, such as bipolar disorder, could have persisted in the population at a relatively high rate of about 2–4% is among the leading questions of evolutionary epidemiology (21, 22). According to Darwinian Theory, common, positively selected traits provided an evolutionary advantage, but in the case of some traits, left almost all members of a population vulnerable to the disease (Figure 1) (23). Supporting evidence has come from comparisons of the human genome to the genome of the chimpanzee, which revealed evidence for positive selection in the opioid receptor genes (24) and immune response genes (25, 26). These studies provided support for a link between entire genes or even gene families and common human traits, such as creativity and novelty seeking, which might have not only provided an evolutionary advantage but also made all humankind susceptible to addiction and other psychiatric disorders (27).

On the other hand, diseases are thought to be subject to negative selection. Only in rare cases has evolutionary selection seemed to have led to the accumulation of Mendelian disorders. An often cited text-book example for a disease with evolutionary advantage is sickle cell anemia, a Mendelian disorder with a population frequency of up to 0.16% in African Americans. Heterozygous mutations in a single disease-causing gene have provided a protective effect against malaria, a common environmental threat in Africa, leading to higher allele frequencies for the protective allele than expected based on mutation rates alone (28–30). However, examples of other disorders have not supported the theory of evolutionary advantages of common variants. The Mendelian disorder cystic fibrosis has reached relatively high prevalence in the population, but increased vulnerability to mutation at a specific location in the disease-causing gene, and not evolutionary advantage, likely contributed to the increased allele frequency of the disease-causing CFTRΔF508 mutation (31). Since more than 1,000 rare mutations in other parts of the gene have been identified as disease-causing alleles, an evolutionary advantage of a single mutation appears to be less likely. Last, but not least, population bottleneck could have resulted in disease aggregation in certain populations. For example, Tay–Sachs disease is a genetically heterogeneous Mendelian disorder with an increased prevalence of 0.04% in the Jewish population (32). The disease is caused by more than 30 different mutated alleles, but because of population isolation and selective mating, the disease could increase in prevalence. These examples demonstrate that disease-causing alleles are relatively rare, even in relatively common diseases and that, with a few exceptions, evolutionary advantages do not explain the increased population prevalence of severe disorders.

## The “Common Disease–Common Variant” Hypothesis

Even though the rare nature of disease-causing mutations had been well accepted by Mendelian geneticists, genetic epidemiologists had been puzzled by the frequent occurrence of common disorders in the population, and also by the discovery of millions of common genetic polymorphisms across the genome that were not well explained by Darwinian Theory. It was tempting to claim that common genetic polymorphisms could be linked to common complex disorders (33). In addition, it had been noticed that association analyses in population samples provided increased statistical power over family based linkage analysis (34). Technical advantages in array-based approaches finally paved the way for genome-wide genotyping of common single-nucleotide polymorphisms (SNPs) and association testing with disease. Genome-wide association studies were widely disseminated across clinical and statistical fields, and thousands of publications followed without further questioning the biological foundation of the common disease-common variant hypothesis. Overall, these studies have revealed a complex genetic structure influencing almost all examined traits and disorders, including bipolar disorder (35–38), but the functional consequences of the common variants remained mostly elusive (39, 40). Overall, the results of these studies have not supported the assumption of a common genetic disease-causing risk factor in bipolar disorder or a link to positive adaptation. Instead, evidence is accumulating that founder effects and drift, but not Darwinian selection, might have caused common allele frequency variability, and a causal link between common variants and common disorders has not been substantiated for most disorders (21, 41, 42).

KEY CONCEPT 5. Common complex disorders Some common medical conditions, such as diabetes or high-blood pressure, are believed to be caused by genetic and environmental factors. Therefore, the transmission in families might not follow a simple Mendelian mode of transmission. According to this model, an individual might not manifest the disease, even though he or she carries a risk mutation, if the environmental exposure has not occurred.

## Alternatives to the Common Disease–Common Variant Hypothesis

**Figure 2:**
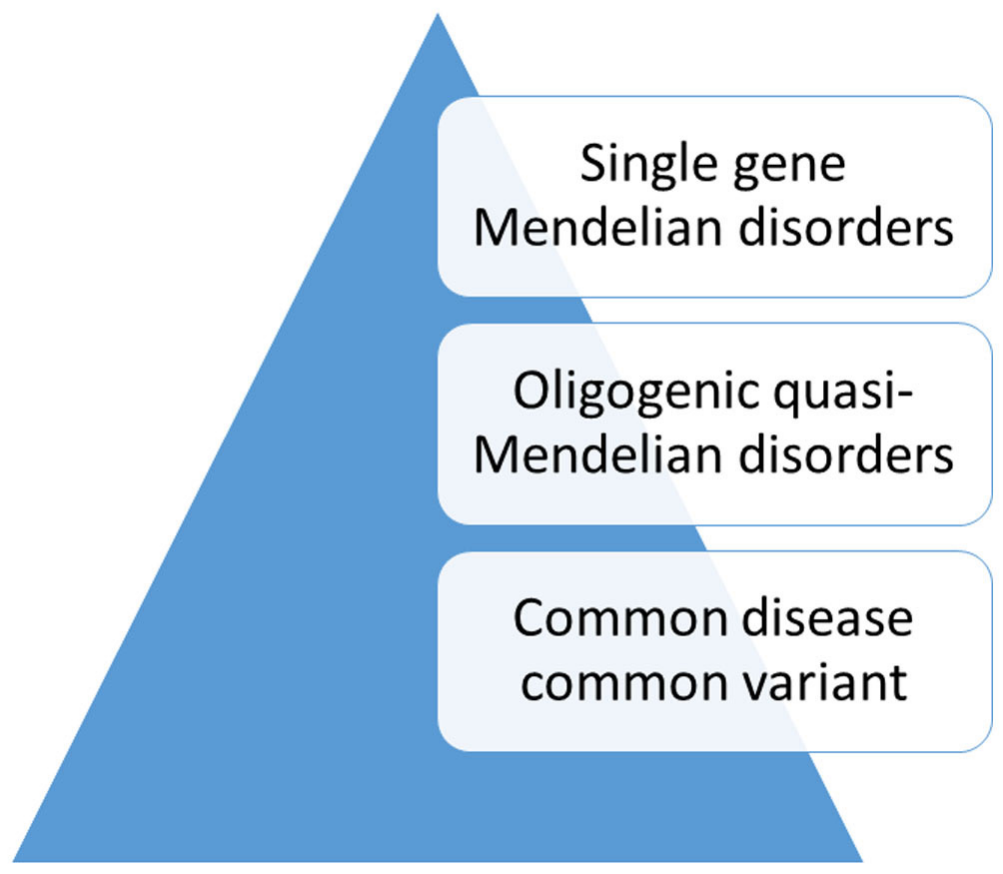
**Genetic models of disease transmission in bipolar disorder**.

While a model of non-random, natural selection had dominated the search for genetic risk factors in traits that might have been related to psychiatric disorders, alternative explanations had also been considered (Figure 2). One hypothesis that had gained attention proposed a more complex polygenic, or even multifactorial, model of transmission (43). An example of polygenic transmission is eye color, which seems to be a purely genetic trait. According to this model, random mutations in many genes, some of which with a dominant effect, influence the expression of the trait in the population (44, 45). On the other hand, height is a trait that is influenced by a complex interplay of genetic and environmental factors (46). An adaptation of this model to psychiatric disorders was the liability threshold model (47). According to this theory, liability to psychiatric disorders or “traits” related to susceptibility follows a continuous distribution in the population. However, this model was contradicted by the finding that family risk did not follow this pattern (48–50). Furthermore, in many families, the disease was transmitted through the paternal and the maternal lineage. This pattern of transmission, also known as assortative mating, contributed to the aggregation of risk factors in a few families with multiple affected family members, whereas in most families the risk was low (51). In general, family studies have not supported a multifactorial threshold model of disease for bipolar disorder (52, 53). Instead, mathematical model fitting in families with bipolar disorder suggested an oligogenic, quasi-Mendelian mode of inheritance with significant locus heterogeneity (54).

## Closing the Circle or is it a Spiral?

**Figure 3:**
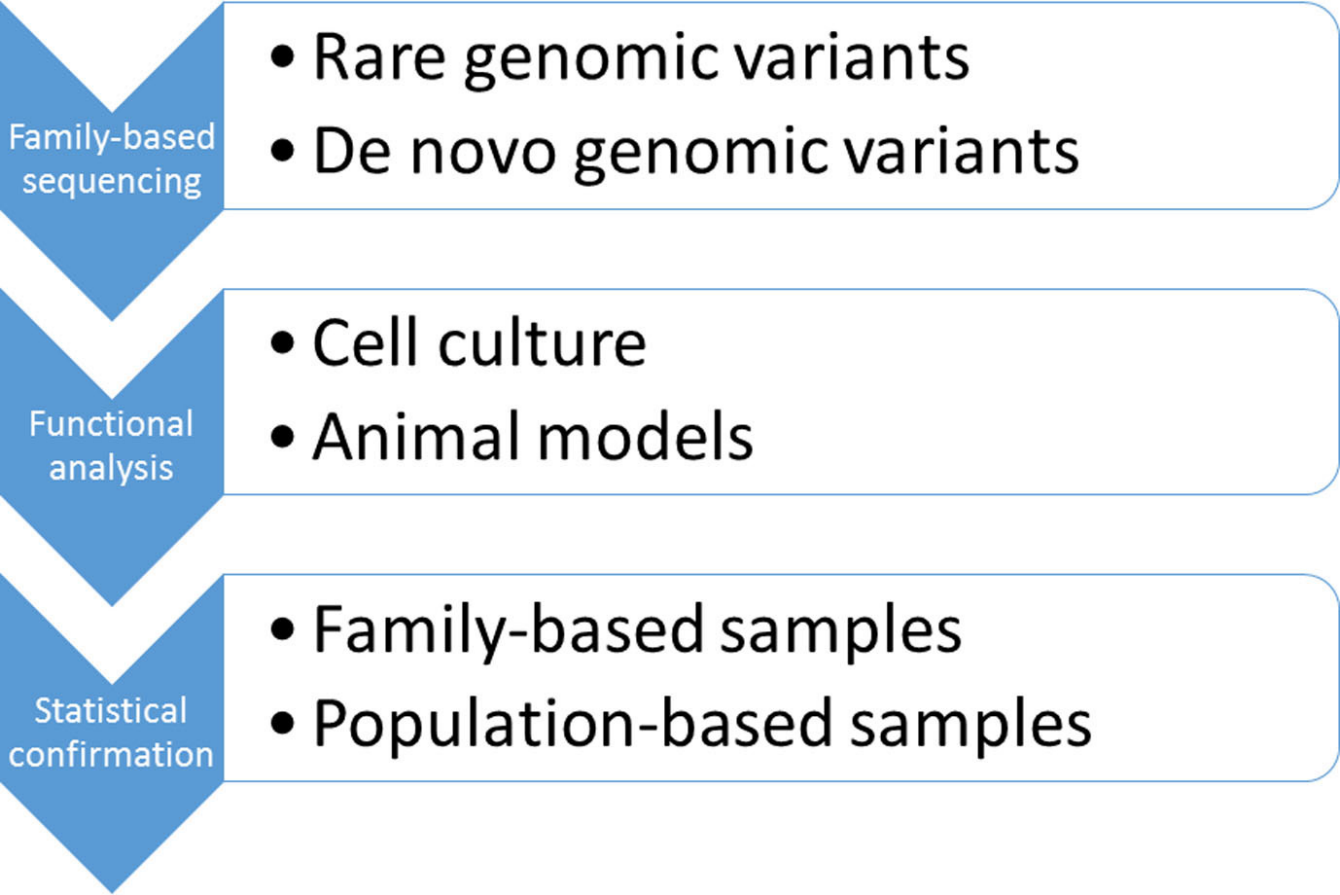
**Identification and confirmation of genetic risk variants in bipolar disorder**.

In 1990s, when large, multi-generational families were first studied, neither the analytical tools nor the biological knowledge were available to solve the problem of a complex, oligogenic inheritance. However, with almost complete information on the human genome available and rapidly falling sequencing costs, the time seemed to be right to revisit disease models proposed more than 20 years ago. Since bipolar disorder is inherited in families, pedigrees seemed to be a natural choice to test the hypothesis of a quasi-Mendelian, oligogenic model of disease transmission (Figure 3). According to this model, it was expected that a few rare and likely functional variants were shared among the affected family members with both parents contributing to the disease risk. Likewise, unaffected family members would not carry the damaging variants. Not only to avoid biases that could be introduced by selection of candidate genes but also to keep the focus on gene-coding regions for which functional information was available, we favored an exome-wide sequencing approach (55). The results of our study suggested that multiple, very rare, and likely protein-damaging mutations in highly conserved gene regions had affected genes that were linked in a single pathophysiological pathway regulated by MAP kinases. All mutations in this family had likely affected a specific signaling pathway known to be involved in the response to mood stabilizing medications. This finding supported the oligogenic hypothesis of genetic risk in bipolar disorder. While a statistical proof of disease association will require larger data sets, these results, nevertheless, point to genes and signaling pathways in which the functional consequences of the mutations could be tested in cell culture and animal models (Figure 3). Since the data have been published, several groups have completed candidate gene sequencing in population and family samples, and exome-wide sequencing in Old Order Amish families (40, 56–58). These studies have found significant haplotype and locus heterogeneity, and further rejected the hypothesis of a single major risk gene for bipolar disorder.

While exome-wide and genome-wide sequencing studies in bipolar disorder are still rare, we have tried to find further support for our hypothesis in studies on a potentially related psychiatric disorder, schizophrenia. These studies have revealed a high degree of *de novo* mutations and rare protein-damaging genomic variants in patients with schizophrenia (59–69). The largest exome-wide study available to date is a population-based Swedish study of 5,079 cases and controls (70, 71). The results of this study supported the hypothesis of significant locus heterogeneity in schizophrenia. Despite an increased burden of potentially damaging rare mutations in cases, no locus-specific associations reached genome-wide statistical significance. The apparent controversy that bipolar disorder appears to be quasi-Mendelian in families, but still very common in the population, could be due to rare mutations in hundreds or thousands of contributing genes, a disease model that has become apparent in intellectual disability and autism (72). Therefore, it will be essential to collect and annotate all identified genetic variants in psychiatric patients and to create a comprehensive searchable database to facilitate genetic testing and personalized genomic medicine (73).

## Where to go from Here?

In bipolar disorder and schizophrenia, increasing evidence supports the role of rare, disease-causing mutations in brain-expressed genes. As a single major risk gene has become highly unlikely, the need for new analytical and statistical approaches has grown. Locus heterogeneity and private mutations challenge hypothesis testing with established statistical methods. However, even for statistically significant associations, the translation into clinical applications will ultimately require the demonstration of biological significance. So far, genome-wide approaches have only scratched the surface of genomic variability. Rare mutations in gene-coding regions certainly constitute only the tip of the iceberg and do not capture the full spectrum of potential disease-causing genomic changes. For example, researchers have only begun to explore the vast functional diversity of non-coding DNA. Functional exploration of micro RNAs (miRNAs), small nuclear RNAs (snoRNAs), and long non-coding RNAs (lncRNAs) could reveal their role in pathway regulation and other cellular processes (74). Furthermore, a growing number of investigators have requested a balanced approach to DNA-based and protein-based studies (75). The field of proteomics has already uncovered the complexities of context-specific and cell-type-specific protein function in a complex network of potential interactions. Posttranslational modifications and their consequences on the structure, function, and intracellular location of the modified proteins provide ever increasing possibilities of variability and interaction. Disease mechanisms might also involve cell-derived membrane vesicles (CVM), which play a role in cell–cell communication. Interdisciplinary approaches will be necessary to clearly elucidate the functional consequences of mutations and protein modifications in the context of intracellular events and pathways, as well as cell networks and developmental processes.

## Summary and Recommendations

To tackle the complexity of psychiatric disorders, we will need a balanced and broad approach to biological and social risk factors in which competing and complementary ideas could receive equal financial support. In a scientific culture that is reflected in phrases, such as “Go big or go home,” the focus is on large heterogeneous population-based samples. While this approach might be useful for studying common traits that are shared by most members of a population, it is less suitable for diseases that are transmitted in families and in which each family may carry a unique combination of susceptibility genes. Rare genomic risk factors with moderately strong effect could be best approached through exome-wide or genome-wide sequencing of multi-generational families in which the disease is transmitted in a Mendelian or “quasi-Mendelian” mode. Based on recent results, it should be recognized that these approaches are at least complementary in studies of schizophrenia and other neuropsychiatric disorders. Rigorous hypothesis testing and rejection of unsupported ideas, as well as transparency and replication of results, will ultimately lead to progress in our understanding of disease processes and risk factors. Reporting of positive, as well as negative, results will increase transparency and reduce redundancy of efforts.

## Conflict of Interest Statement

The author declares that the research was conducted in the absence of any commercial or financial relationships that could be construed as a potential conflict of interest.
